# Exercise-Induced Acid–Base, Electrolyte, and Metabolic Responses in Colombian Creole Horses Across Different Gait Modalities

**DOI:** 10.3390/ani16172738

**Published:** 2026-09-02

**Authors:** Angélica María Zuluaga-Cabrera, José Eduardo Escobar-Riomalo, María Fernanda Giraldo-Arboleda, Juan Manuel Morales-Castañeda, Guilherme Barbosa da Costa, Iván Darío Martinez, Yasser Y. Lenis, María Patricia Arias-Gutiérrez

**Affiliations:** 1 Grupo de Investigación en Salud y Cuidado Animal (GISCA), Faculty of Zootechnics and Veterinary Medicine, Institución Universitaria Visión de las Américas, Medellín 050031, Colombia; jose.escobar@uam.edu.co (J.E.E.-R.); maria.giraldoar@uam.edu.co (M.F.G.-A.); 2 Equine Sport Medicine Master Program, Veterinary Faculty, Universidad de Córdoba, 14014 Córdoba, Spain; juan601992@hotmail.com; 3 Veterinary Medicine School, Barretos Educational Foundation University Center—UNIFEB, Barretos 14784-400, Brazil; guilherme.b.costa@unesp.br; 4 Faculty of Agricultural and Veterinary Sciences—FCAV, São Paulo State University Júlio de Mesquita Filho—UNESP, Jaboticabal Campus, Jaboticabal 14884-900, Brazil; 5 Faculty of Agricultural Sciences, Animal Welfare and Ethology Specialization, Fundación Universitaria Agraria de Colombia (UNIAGRARIA), Bogotá 111321, Colombia; martinez.ivan1@uniagraria.edu.co; 6 One Health Veterinary Research Initiative (OHVRI), School of Veterinary Medicine, Faculty of Agrarian Science, Universidad de Antioquia, Medellín 050021, Colombia; yasser.lenis@udea.edu.co; 7 Investigación en Ciencias Animales (INCA) Research Group, Faculty of Agricultural and Natural Sciences, CES University, Medellín 050021, Colombia; marias@ces.edu.co

**Keywords:** exercise-induced acidosis, hemogasometry, lactate

## Abstract

Exercise physiology in Colombian Creole Horses remains poorly characterized despite the breed’s extensive use in athletic activities. The objective of this study was to evaluate exercise-induced changes in blood biochemical and gas parameters and to determine whether these responses differed according to sex, geographic origin, or gait modality. An analytical observational study with repeated measures was conducted in 76 athletic CCHs. Blood samples were obtained from the jugular vein at rest and immediately after exercise to measure acid–base indicators (pH, carbon dioxide pressure, bicarbonate), electrolytes (sodium, potassium, calcium, chloride), and metabolic variables (glucose, lactate, creatinine). Within-subject comparisons were performed to quantify exercise-induced changes, and subgroup analyses were conducted by sex, geographic region, and gait modality. Exercise resulted in physiologically consistent responses, including a mild reduction in pH (−0.01), decreases in carbon dioxide pressure (−2.39 mmHg) and bicarbonate (−1.7 mmol/L), and a moderate increase in lactate (+2.96 mmol/L), indicating exercise-associated metabolic acidosis and anaerobic metabolism. Glucose and creatinine also increased modestly, while electrolyte concentrations remained relatively stable. No meaningful differences were detected between sexes or geographic regions. As expected, gait modality significantly influenced acid–base responses, with horses performing *trocha* exhibiting greater reductions in carbon dioxide pressure and bicarbonate. These findings indicate that gait type modulates the magnitude of exercise-induced acid–base responses in Colombian Creole Horses.

## Simple Summary

Physical exercise produces physiological changes in athletic horses that help the body meet the energetic demands of movement. However, limited information exists about these responses in Colombian Creole Horses, a breed widely used in sport competitions. This study evaluated how exercise affects blood chemistry and acid–base balance in 76 Colombian Creole Horses and investigated whether these changes differ according to sex, geographic origin, or gait modality. Blood samples were collected at rest and immediately after exercise to measure indicators related to metabolism, electrolytes, and acid–base status. Exercise produced expected physiological responses, including increased blood lactate and glucose levels and mild changes in acid–base balance, indicating activation of energy reserves and anaerobic metabolism during exertion. Electrolyte levels remained relatively stable, suggesting effective physiological regulation in these trained animals. Differences between males and females and between geographic regions were minimal. However, the type of gait (modality) performed by the horses influenced the magnitude of some physiological responses. Horses performing the *trocha* gait showed greater alterations in acid–base responses than those performing other gaits, suggesting that different gaits impose different physiological demands. These findings improve understanding of exercise physiology in Colombian Creole Horses (CCHs) and may help veterinarians, trainers, and owners design training strategies that support performance and the welfare of equine athletes.

## 1. Introduction

Exercise induces changes in acid–base status, fluid balance, and electrolyte homeostasis that are proportional to the intensity and duration of physical activity. However, the magnitude of these physiological responses is influenced not only by exercise intensity and duration but also by locomotor biomechanics, muscle recruitment patterns, and locomotor–respiratory interactions [1].

The Colombian Creole Horse (CCH) is an athletic breed selectively developed for four distinct gait disciplines (*paso fino, trocha, trocha & gallop, and trot & gallop*), each characterized by specific stride mechanics, limb coordination, and movement patterns. These horses perform moderate- to high-intensity exercise requiring rapid, repetitive limb movements and substantial anaerobic energy production. Consequently, they undergo systematic athletic training comparable to that of other equine performance disciplines.

Gait is the defining athletic characteristic of the Colombian Creole Horse and underpins performance in this breed. During training and competition, horses perform moderate- to high-intensity exercise characterized by rapid, repetitive limb movements and substantial anaerobic energy demand. The four official gait disciplines differ in stride mechanics, limb coordination, muscular activation, and locomotor–respiratory coupling, factors that can influence oxygen consumption, carbon dioxide elimination, and overall metabolic demand during exercise [2,3]. Consistent with these characteristics, previous studies in Colombian Creole Horses have reported gait-related differences in oxygen consumption, respiratory responses, and exercise intensity [4,5]. Similar associations between locomotor mechanics and metabolic efficiency have also been described in other equine athletes [6].

Blood gas variables, acid–base status, electrolytes, lactate, and glucose are widely used to characterize the physiological response to exercise and to assess metabolic stress in athletic horses [7]. Although exercise-induced metabolic acidosis and increases in blood lactate are expected during moderate- to high-intensity exercise, little is known about whether the magnitude of these responses differs among the gait disciplines of the Colombian Creole Horse. Likewise, the potential influence of sex and geographic location on these physiological responses has received little attention.

Therefore, the aim of this study was to evaluate exercise-induced changes in venous blood gas, biochemical, and electrolyte variables in Colombian Creole Horses and to determine whether these responses differ according to gait discipline, sex, and geographic location. We hypothesized that exercise would be associated with an increase in lactate and mild acid–base changes by gait modality, and it could be associated with differences in pCO_2_ and bicarbonate responses. Comparisons by sex and geographic origin were considered secondary exploratory analyses without a prespecified directional hypothesis.

## 2. Materials and Methods

### 2.1. Study Population and Design

The study included clinically healthy CCHs (physical examination, a CBC and serum biochemistry profile were performed to exclude subclinical disease in every horse) actively competing in the official gait modalities (*paso fino, trocha, trocha & gallop*, and *trot & gallop*). Horses were recruited from equestrian facilities located in four Colombian departments representing three tropical environmental regions. Because this was an observational field study involving horses presented for routine sports medicine evaluation, a convenience sample was used. Consequently, no a priori sample size calculation was performed. Horses from Córdoba and Valle del Cauca were located in the low tropical region (0–50 m above sea level [masl]; 0–196 ft; n = 35), horses from Antioquia in the middle tropical region (1450 masl; 4757 ft; n = 38), and horses from Cundinamarca in the high tropical region (2450–3100 masl; 8038–10,170 ft; n = 3). Because these regions differ in altitude and associated environmental conditions, geographic location was included as a potential source of physiological variation. The study population included horses aged 7.5 ± 4.94 years with a body condition score of 8/9 according to the Henneke scoring system and a mean body weight of 370.7 ± 21.5 kg. All horses were in regular competition and had maintained their routine training program (5–6 days/week) for at least six months before the study and were managed under comparable husbandry conditions, including routine athletic training, housing, feeding, and preventive veterinary care.

Inclusion criteria comprised horses that: (i) were actively training and competing for at least six months before the study; (ii) were considered fit for competition by their owners and trainers; (iii) were clinically healthy based on a physical examination performed before exercise; and (iv) completed both resting and post-exercise sampling. Horses showing evidence of systemic illness, musculoskeletal lameness, or any clinical condition that could impair exercise performance were excluded. Only non-pregnant females and sexually intact males were included to minimize physiological variability associated with reproductive status.

Horses were evaluated under their usual management conditions during routine competition or training sessions. Competitions were performed according to the regulations of each gait discipline and lasted approximately (20–25 min). Resting blood samples were obtained before exercise while horses were quietly standing. Post-exercise blood samples were collected immediately after completion of the exercise (within 2 min) to characterize the acute physiological response. Rectal temperature was measured immediately after blood collection.

The study design, conduct, and reporting followed the STROBE Statement for observational studies (https://www.strobe-statement.org/) to ensure transparency in participant selection, variable definition, and analysis.

### 2.2. Exercise Protocol and Data Collection

The exercise protocol consisted of the horses performing their routine competition or training exercise according to their respective gait discipline under field conditions. The intensity and duration of exercise reflected the normal athletic demands of each discipline and were not experimentally modified. The exercise protocol was classified as moderate-intensity submaximal exercise, with an immediate post-exercise measurement of lactate concentration of 3.62 IQR [2.20–4.39 mmol/L] and a maximal heart rate of 204 ± 23 bpm IQR [193–215 bpm], corresponding to approximately 93% of the expected maximal heart rate (theoretical maximal heart rate for CCHs of 220 bpm) [1,2]. These findings indicate a near-maximal cardiovascular demand accompanied by moderate-to-high anaerobic metabolic activation. Samples were collected immediately after completion of the exercise to evaluate the acute physiological response to exercise.

Blood samples (1.5 mL) were collected by jugular venipuncture into lithium–heparin syringes under two physiological conditions (at rest and immediately after exercise). Post-exercise rectal temperature (°C) was also recorded.

Blood gas and biochemical analyses included acid–base variables (pH, partial pressure of carbon dioxide [pCO_2_], and bicarbonate [HCO_3_^−^]), electrolyte concentrations (sodium [Na^+^], potassium [K^+^], calcium [Ca^2+^], and chloride [Cl^−^]), and metabolic variables (glucose, lactate, and creatinine). Hematocrit was also determined. Samples were analyzed immediately using the epoc^®^ Blood Analysis System (Siemens Healthineers, Munich, Germany). The epoc system is a point-of-care platform that uses a single-use sensor card and electrochemical detection. In brief, pH, pCO_2_, and electrolytes are determined using potentiometric/ion-selective sensor principles, while metabolites such as glucose and lactate are measured using amperometric enzymatic sensors; bicarbonate is calculated from measured pH and pCO_2_ according to the Henderson–Hasselbalch relationship. The measured variables included pH, pCO_2_, HCO_3_^−^, Na^+^, K^+^, ionized Ca^2+^, Cl^−^, glucose, lactate, and creatinine.

The epoc analyzer has been evaluated against laboratory blood-gas systems using equine samples, with parameter-dependent agreement. Published equine evaluations support its use as a point-of-care device while cautioning that results for some analytes, including pCO_2_ and ionized calcium, may not be interchangeable with those from other analyzers [8,9,10]. Because this was a field study, ambient temperature, altitude-related operating conditions, and other environmental influences on analyzer performance were not experimentally assessed. All samples within a horse were analyzed using the same device platform to reduce method-related variation in the paired comparison. Strong Ion Difference (SID) was calculated using Stewart’s methodology for gait modality stratification [11].

The explanatory variables recorded for each horse included sex (male or female), gait discipline (*trocha, trocha & gallop, trot & gallop, or paso fino*), geographic location (Antioquia, Valle del Cauca, Córdoba, or Cundinamarca), and altitude of the evaluation site. The principal outcome variables were the changes in acid–base status, electrolyte concentrations, metabolic biomarkers, hematocrit, and rectal temperature between the resting and post-exercise conditions.

### 2.3. Statistical Analysis

Data were analyzed in Stata 19.5 (StataCorp, College Station, TX, USA). Data completeness, paired animal identifiers, and concordance of sex, geographic origin, and gait modality between pre- and post-exercise records were verified before analysis. 

Normality of the distribution of each of the ten quantitative variables (pH, pCO_2_, HCO_3_^−^, Na^+^, K^+^, Ca^2+^, Cl^−^, glucose, lactate and creatinine) was assessed with the Shapiro–Wilk test, applied both to the overall sample and within each level of every categorical factor (sex, geographic location, gait modality); a variable was considered to satisfy normality for a given factor only when all of its levels yielded *p* > 0.05. Homogeneity of variance across groups was evaluated with Levene’s test.

The exercise-induced effect on each variable was assessed with a paired-samples *t*-test when the pre–post difference met the normality assumption, or with the Wilcoxon signed-rank test otherwise. Ninety-five percent confidence intervals (95% CI) for the mean/median pre–post difference were obtained analytically (Student’s t distribution) for parametric tests and via a non-parametric bootstrap (5000 resamples, percentile method) for the Wilcoxon test.

For the two-level factor (sex), an independent-samples Welch’s *t*-test or Mann–Whitney U test was applied, according to the normality/homogeneity-of-variance criteria described above; 95% CIs for the parametric mean difference were obtained analytically, and for the non-parametric median difference via bootstrap (5000 resamples). For the four-level factors (geographic location, gait modality), a one-way ANOVA or Kruskal–Wallis test was applied accordingly, followed, when the omnibus test was significant, by Tukey’s honestly significant difference (HSD) post hoc test (parametric) or Dunn’s post hoc test with Bonferroni correction for multiple comparisons (non-parametric). A two-tailed *p*-value < 0.05 was considered statistically significant throughout.

Because each horse contributed only two repeated measurements (before and after exercise), exercise-induced responses were analyzed using paired within-subject procedures. Group comparisons were subsequently performed using the individual change (Δ) values, ensuring statistical independence among observations used for between-group analyses. Given the observational field design and the presence of only two repeated measurements per subject, this analytical approach was considered more parsimonious than mixed-effects modelling while adequately addressing the predefined study hypotheses.

## 3. Results

### 3.1. Study Population Characteristics

A total of 76 Colombian Creole Horses (CCHs) with complete paired measurements obtained at rest and immediately after exercise were included in the analysis. Horses were evaluated following requests from their owners or riders for assessment by a private equine sports medicine veterinarian between 2022 and 2025. All enrolled horses had complete pre- and post-exercise datasets; therefore, no missing or incomplete paired observations were identified.

All horses were maintained under routine management conditions at privately owned equine facilities. Animals were housed in covered outdoor stalls and received a standard diet consisting of Angleton/Pangola hay, alfalfa pellets, a commercial balanced concentrate, and a mineral salt supplement. Fresh water was available ad libitum under routine management before and after exercise, but access and actual intake during the training bout were not standardized or quantified. Informed consent for the participation of each horse was obtained from its owner before enrollment.

The study population included both sexes, comprising n = 39 males (51.3%) and n = 37 females (48.7%), with all females confirmed as non-pregnant and all males sexually intact, minimizing physiological variability associated with reproductive status. All animals were high-performing individuals within their respective categories and were actively competing, with some ranked among the top athletes in their gait modality in Colombia.

Horses were distributed across multiple geographic regions of Colombia, predominantly from Antioquia (n = 38) and Valle del Cauca (n = 34), with smaller representation from Cundinamarca (n = 3) and Córdoba (n = 1). Horses from Cundinamarca and Valle del Cauca were retained for descriptive purposes only and were excluded from inferential comparisons.

Regarding gait modality, individuals were classified as *trocha* (n = 36), *paso fino* (n = 23), *trocha & gallop* (n = 9), and *trot & gallop* (n = 8). 

### 3.2. Baseline (Resting) Biochemical Profile

Baseline (resting) biochemical and blood gas parameters for the study population are summarized in Table 1. Overall, values were within expected physiological ranges for athletic horses at rest [10]. 

### 3.3. Exercise-Induced Changes

Exercise-induced changes in biochemical and blood gas parameters are summarized in Table 1. Exercise induced a consistent metabolic response. Lactate increased from 0.76 mmol/L [0.65–0.81] at rest to 3.62 mmol/L [2.20–4.39] after exercise (*p* < 0.001). Glucose and creatinine also increased significantly. Acid–base changes were mild but statistically detectable: pH decreased by a median of −0.011 (*p* = 0.018), pCO2 decreased by −2.4 mmHg (*p* = 0.0016), and HCO_3_- decreased by −1.7 mmol/L (*p* < 0.001). 

### 3.4. Stratified Analysis by Sex and Geographic Location (Region of Origin)

To further explore potential sources of variability in the exercise-induced response, stratified analyses were performed according to sex, gait modality, and geographic region. These analyses aimed to assess whether the magnitude of biochemical changes differed across subgroups of the study population. Exercise-induced changes were first evaluated by sex (Table 2), followed by comparisons across gait modalities (Table 3), and the main geographic regions represented in the study (Table 4).

Exercise-induced changes showed consistent directional patterns across both sexes, with all major analytes responding similarly to physical exertion. Both males and females exhibited a moderate increase in lactate concentrations following exercise, accompanied by decreases in pH and HCO_3_^−^.

Although minor differences in magnitude were observed. Males showed a slightly higher mean increase in lactate, suggesting a potentially greater anaerobic response, whereas females exhibited a marginally greater reduction in pH. 

From an inferential perspective, no statistically significant differences were identified between sexes for most analytes, including lactate, pH, HCO_3_^−^, and glucose (*p* > 0.05).

Overall, these findings suggest that sex has a limited influence on the acute biochemical response to exercise in CCHs under comparable management conditions and exercise intensity.

Overall, all gait modalities exhibited consistent directional responses to exercise, including increased lactate and glucose concentrations and reductions in pH and HCO_3_^−^, reflecting a generalized pattern of exercise-induced metabolic compensated changes.

However, significant differences were identified in parameters related to acid–base balance. Specifically, the magnitude of change in pCO_2_ differed significantly across gait modalities (post hoc analysis in Supplementary Table S3), with *trocha* horses showing the greatest decrease (−4.38 mmHg), whereas *paso fino* horses exhibited a slight increase (+0.25 mmHg). Similarly, changes in HCO_3_^−^ also differed significantly between groups (*p* = 0.008) (post hoc analysis in Supplementary Tables S2 and S4), with *trocha* horses showing the largest reduction (−3.50 mmol/L), in contrast to the minimal decrease observed in *paso fino* horses (−0.7 mmol/L) (Supplementary Table S1). 

These findings suggest that *trocha* is associated with a more pronounced metabolic and respiratory response to exercise, compatible with higher anaerobic demand. In contrast, *paso fino* appears to be associated with a more stable acid–base profile, potentially reflecting greater metabolic efficiency or lower exercise intensity.

For the remaining variables, including lactate and electrolytes, no statistically significant differences were observed across gait modalities (*p* > 0.05). Nevertheless, descriptive trends indicated higher lactate accumulation in *trocha,* and *trot & gallop* horses compared to *paso fino*, although variability within groups was considerable.

Overall, while the general physiological response to exercise is conserved across gait types, these results indicate that gait modality may influence the magnitude of acid–base responses, particularly in relation to respiratory and buffering mechanisms. 

To determine whether exercise-induced acid–base changes reflected a respiratory/metabolic disturbance rather than an underlying strong ion (electrolyte) imbalance, the simplified Stewart SID ([Na^+^] + [K^+^] + [Ca^2+^] − [Cl^−^], mmol/L) was compared among gait modalities at baseline, post-exercise, and for the exercise-induced change (Δ), using ANOVA or the Kruskal–Wallis test according to normality. SID did not differ significantly among modalities at any time point (baseline: *p* = 0.415; post-exercise: *p* = 0.215; Δ: *p* = 0.613), so no post hoc tests were performed. This stability of the strong ion balance suggests that the greater post-exercise decline in HCO_3_^−^ and pCO_2_ previously observed in *Trocha* versus *Paso fino* reflects a respiratory/metabolic mechanism rather than an electrolyte-driven disturbance.

In the geographic stratified analysis, no statistically significant differences were identified in the magnitude of exercise-induced changes between geographic regions for any of the evaluated analytes (*p* > 0.05 for all comparisons) (Table 5).

Overall, the physiological response to exercise was comparable across regions, indicating that, under similar training and management conditions, geographic location (Antioquia and Valle del Cauca) is unlikely to have a substantial effect on the acute biochemical response to exercise in CCHs. 

## 4. Discussion

One of the most relevant findings of this study is that gait modality influenced exercise-induced acid–base responses. Specifically, horses performing *trocha* gait exhibited greater reductions in both pCO_2_ and HCO_3_^−^, consistent with a more pronounced metabolic acidosis-tendency compatible with respiratory compensation. In contrast, *paso fino*, *trocha & gallop* and *trot & gallop* horses showed minimal changes in HCO_3_^−^ and even a slight increase in pCO_2_, suggesting a more stable acid–base status during exercise. These findings indicate that different gait modalities may impose distinct physiological demands, likely reflecting differences in biomechanical efficiency, muscular recruitment, and exercise intensity. This supports the notion that not all gaits are physiologically equivalent, even within the same breed and competitive context.

Although formal interaction models were not applied, the combined stratified analyses suggest that exercise-induced physiological responses are influenced by gait modality emerging as the most relevant determinant. Significant differences in pCO_2_ and HCO_3_^−^ responses indicate that *trocha* horses experience a more pronounced metabolic and/or respiratory challenge during exercise. This effect is likely related to the higher effort and physiological demand associated with *trocha*, the faster gait [5], which impose greater cardiovascular, respiratory, and metabolic demand. A greater magnitude of physiological stress is observed in *trocha* horses, similar to that reported in humans performing high-speed or explosive sports [12]. 

As expected, lactate concentrations increased moderately following exercise in all horses, confirming the activation of anaerobic metabolism during physical exertion, as previously described in previous studies in Colombian creole horses [3,7]. However, no statistically significant differences were observed across gait modalities, despite descriptive trends suggesting higher lactate accumulation in *trocha* and *trot & gallop* horses. In addition, creatinine increased modestly from 1.19 ± 0.19 to 1.34 ± 0.22 mg/dL, corresponding to an approximately 12.6% increase. Given the acute post-exercise timing of sampling, this mild change was interpreted as most likely reflecting a transient pre-renal response associated with exercise-related fluid shifts rather than a broader metabolic adaptation. Overall, lactate and creatinine responses should therefore be interpreted as acute exercise-associated changes rather than evidence of gait-specific metabolic adaptation. 

Electrolyte concentrations remained relatively stable following exercise, with only minor changes observed in Na^+^, K^+^, Ca^2+^, and Cl^−^. This stability suggests effective homeostatic regulation in response to acute physical exertion. The absence of clinically relevant electrolyte disturbances indicates that the horses included in this study were well adapted to exercise conditions and maintained adequate physiological balance despite metabolic stress. This finding is consistent with previous reports in athletic horses and reinforces the importance of conditioning and management in maintaining electrolyte equilibrium.

According to Stewart’s physicochemical approach, SID remained stable across gait modalities at rest and after exercise, with similar exercise-induced changes among groups. In contrast, *trocha* horses showed greater post-exercise decreases in HCO_3_^−^ and pCO_2_ than *Paso Fino* horses. These findings suggest that the greater acid–base response observed in *trocha gait* was primarily associated with metabolic and respiratory responses to exercise rather than changes in strong ion balance. Thus, gait modality appears to influence the magnitude of the exercise-induced acid–base response without significantly affecting its strong ion component.

Sex did not appear to significantly influence the magnitude of exercise-induced biochemical changes. Both males and females exhibited similar patterns of lactate increase, acid–base disturbance, and metabolic adaptation. These results suggest that, under comparable training and management conditions, the acute physiological response to exercise is largely independent of sex, supporting the use of mixed-sex populations in similar experimental designs. In contrast to findings in other equestrian disciplines such as eventing, where differences in competitive performance have been reported among geldings, stallions, and mares—differences that are also influenced by factors such as training level, conditioning status, and management practices—[13], such variations are not observed in CCHs. This is consistent with broader evidence indicating that, unlike in humans, sex does not appear to have a major influence on physical performance in dogs and horses. This is largely because, in humans, there are marked morphometric and physiological differences between males and females—such as greater muscle mass, hemoglobin concentration, and aerobic capacity in males—which can significantly impact athletic performance [14]. In contrast, these differences are much less pronounced in most domestic animal species, resulting in a more comparable performance capacity between sexes. Additionally, in horses and dogs, performance is more strongly influenced by factors such as training level, discipline, genetics, and management rather than sex alone [14]. 

No statistically significant differences were identified between geographic regions in any of the evaluated parameters. Nonetheless, environmental factors such as altitude and climate should not be completely disregarded and deserve further investigation under more controlled experimental conditions. Consistent with previous studies conducted in Colombia, which included horses from locations ranging from below 1000 to over 2500 m above sea level, the present study found no evidence that geographic location significantly affected the acute biochemical response to exercise [10].

Previous studies have reported differences in locomotor–respiratory coupling among Colombian creole horse’ gaits, explaining metabolic and respiratory differences influencing pH changes (p.e. pCO_2_), as reported by several authors, although these horses belong to the same breed, they originate from distinct genetic lines, which further contributes to their morphological, functional, and performance differences [15]. A stride frequency of approximately 2.8 strides·s^−1^ has been described for the *paso fino* gait, with a stride frequency-to-breathing ratio of 2.0 [4]. In contrast, stride frequency has been reported as 2.3 ± 0.2 strides·s^−1^ during *trot* and *trocha*, and 2.2 ± 0.1 strides·s^−1^ during gallop. The corresponding stride frequency-to-breathing ratios were 1.7:1 (±0.6) for *trot* or *trocha* and 3:2 (±0.3) during gallop [5]. In the present study, the differences in pCO_2_ and HCO_3_^−^ between resting and post-exercise measurements indicated a mild acidifying response during the *trocha* gait. This finding is particularly noteworthy because ventilatory strategies differ among gaits. In this context, *trocha* exhibited the lowest stride frequency-to-breathing ratio, which may suggest a faster ventilatory pattern. Such a pattern could promote altered ventilatory strategy leading to differences in CO_2_ elimination in the bloodstream, potentially contributing to alterations in acid–base balance during exercise. Similar to CCHs, research in Arabian horses has reported changes in metabolic and respiratory variables, such as the respiratory exchange ratio (RER) and oxygen consumption (VO_2_), depending on the gait performed during endurance exercise [6]. These findings indicate that biomechanical characteristics of locomotion can influence metabolic responses during exercise.

Age was not evaluated in the present study, although it may represent an important factor associated with metabolic capacity. Nevertheless, all animals included corresponded to horses trained for high-level competitive performance, and no individuals younger than four years were included. Young, gaited horses have been shown to activate metabolic strategies to sustain exertion, including glycogenolysis leading to increased circulating glucose concentrations [16].

Several limitations should be acknowledged. The sample size was relatively small in some gait and geographic regions categories, which may have reduced the statistical power to detect differences for certain variables. Additionally, direct measurements of exercise intensity, like oxygen consumption (VO_2_), were not obtained. However, these variables have been recently characterized according to gait modality in Colombian Creole horses and may serve as a useful physiological reference for interpreting the observed responses [2,4,5]. Another limitation was that electrolyte supplementation, which could have mitigated the severity of acid–base disturbances in these animals, was not controlled.

### Clinical Relevance

From a practical perspective, these findings indicate that routine post-exercise venous blood-gas interpretation in Colombian Creole Horses should consider gait modality, particularly in *trocha* horses, where larger exercise-induced reductions in bicarbonate and pCO_2_ may represent normal physiological responses rather than evidence of pathological acid–base disturbances. This information may assist veterinarians and trainers in differentiating expected athletic responses from clinically relevant abnormalities during performance evaluations.

## 5. Conclusions

These findings provide reference information for the interpretation of exercise-induced acid–base and biochemical responses in Colombian Creole Horses under field conditions and support the inclusion of gait modality when evaluating exercise physiology in this breed. Unlike laboratory treadmill studies, the present investigation reflects physiological responses under real competitive conditions.

## Figures and Tables

**Table 1:** Metabolic and venous blood-gas variables of the general sample before (T0) and after (T1) exercise (n = 76 horses).

Variable	Pre-Exercise (T0)	Post-Exercise (T1)	*p*-Value	Effect Size
pH	7.41 [7.41–7.42]	7.41 [7.40–7.41]	0.021 *	−0.271 ^a^
pCO_2_ (mmHg)	41.43 ± 3.48	39.04 ± 6.02	<0.001 **	−0.394 ^a^
HCO_3_^−^ (mmol/L)	26.40 [26.00–26.80]	24.70 [23.25–25.65]	<0.001 **	−0.821 ^b^
Na^+^ (mmol/L)	137.00 [137.00–138.00]	138.00 [137.00–138.00]	<0.001 **	0.528 ^b^
K^+^ (mmol/L)	4.00 [3.90–4.10]	3.80 [3.70–3.95]	0.018 *	−0.277 ^a^
Ca^2+^ (mmol/L)	1.60 [1.57–1.64]	1.50 [1.42–1.54]	<0.001 **	−0.735 ^b^
Cl^−^ (mmol/L)	102.00 [102.00–103.00]	102.00 [102.00–103.00]	0.209 ^ns^	−0.193 ^b^
Glucose (mg/dL)	107.50 [104.00–112.00]	118.50 [112.50–123.01]	<0.001 **	0.530 ^b^
Lactate (mmol/L)	0.76 [0.65–0.81]	3.62 [2.20–4.39]	<0.001 **	0.986 ^b^
Creatinine (mg/dL)	1.19 ± 0.19	1.34 ± 0.22	<0.001 **	0.849 ^b^

Normally distributed variables are expressed as mean ± standard deviation and were compared using the paired-samples Student’s *t*-test. Non-normally distributed variables are expressed as median [95% bootstrap confidence interval, 5000 resamples] and were compared using the Wilcoxon signed-rank test for related samples. Effect size calculated as Cohen’s dz ^a^ for paired samples or rank-biserial correlation ^b^. * *p* < 0.05; ** *p* < 0.01. pCO_2_: partial pressure of carbon dioxide; HCO_3_^−^: bicarbonate; ns: not significant.

**Table 2:** Post-exercise biochemical variables stratified by sex (n = 76).

Variable	Females (n = 37)	Males (n = 39)	*p*-Value
pH	7.41 [7.40–7.43]	7.42 [7.40–7.43]	0.909
CO_2_ (mmHg)	41.49 ± 3.29	41.37 ± 3.70	0.882
HCO_3_^−^ (mmol/L)	26.40 ± 2.07	26.53 ± 1.75	0.763
Na^+^ (mmol/L)	137.00 [136.00–138.00]	138.00 [137.00–138.00]	0.166
K^+^ (mmol/L)	3.90 [3.70–4.10]	4.00 [3.80–4.20]	0.127
Ca^2+^ (mmol/L)	1.63 [1.56–1.66]	1.58 [1.52–1.66]	0.286
Cl^−^ (mmol/L)	102.00 [100.00–104.00]	102.00 [102.00–104.50]	0.111
Glucose (mg/dL)	110.03 ± 11.94	107.15 ± 12.60	0.311
Lactate (mmol/L)	0.74 [0.54–0.99]	0.76 [0.59–0.90]	0.940
Creatinine (mg/dL)	1.17 ± 0.22	1.20 ± 0.17	0.473

Comparisons performed using independent *t*-test or Mann–Whitney U test. Normally distributed variables are expressed as mean ± standard deviation and were compared using the paired-samples Student’s *t*-test. Non-normally distributed variables are expressed as median [95% bootstrap confidence interval, 5000 resamples].

**Table 3:** Post-exercise biochemical parameters stratified by gait modality (n = 76).

Variable	Paso Fino (n = 23)	TR (n = 36)	TRG (n = 9)	TG (n = 8)	*p*-Value
pH	7.41 ± 0.05	7.40 ± 0.04	7.40 ± 0.04	7.41 ± 0.02	0.994
CO_2_ (mmHg)	41.30 [38.25–46.00]	37.75 [34.83–42.33]	40.80 [37.60–43.20]	39.75 [35.40–40.80]	0.135
HCO_3_^−^ (mmol/L)	26.00 [24.50–26.75] ^a^	23.25 [20.20–25.92] ^b^	23.40 [22.30–24.70]	24.75 [21.75–25.55]	0.032 *
Na^+^ (mmol/L)	138.00 [137.00–139.00]	138.00 [136.00–139.00]	137.00 [137.00–139.00]	138.00 [136.75–139.00]	0.958
K^+^ (mmol/L)	4.00 [3.65–4.20]	3.75 [3.50–4.10]	3.70 [3.60–3.80]	3.65 [3.58–3.98]	0.455
Ca^++^ (mmol/L)	1.53 [1.40–1.61]	1.47 [1.38–1.55]	1.38 [1.35–1.55]	1.55 [1.38–1.58]	0.293
Cl^−^ (mmol/L)	101.83 ± 2.39	102.03 ± 2.76	103.33 ± 2.69	102.62 ± 1.30	0.447
Glucose (mg/dL)	107.00 [102.00–122.00] ^a^	122.50 [114.75–143.25] ^b^	119.00 [101.00–128.00]	111.50 [104.00–131.50]	0.024 *
Lactate (mmol/L)	2.19 [1.04–4.12]	4.54 [1.66–8.12]	3.14 [2.17–4.21]	3.18 [2.16–6.01]	0.082
Creatinine (mg/dL)	1.32 ± 0.22	1.34 ± 0.21	1.28 ± 0.26	1.45 ± 0.14	0.405

* Comparisons performed using Kruskal–Wallis test. Within each row, values with different superscript letters (^a,b^) differ significantly (*p* < 0.05) according to the corresponding post hoc comparison. Normally distributed variables are expressed as mean ± standard deviation and were compared using the paired-samples Student’s *t*-test. Non-normally distributed variables are expressed as median [95% bootstrap confidence interval, 5000 resamples]. TR = trocha gait, TRG = trocha & gallop gait, TG = trot & gallop gait.

**Table 4:** Simplified strong ion difference (SID) before (T0) and after (T1) exercise, and its exercise-induced change (Δ = T1 − T0), according to gait modality (n = 75; one animal excluded due to a missing Ca^2+^ value).

Time Point	Paso Fino	TR	TRG	TG	*p*-Value
SID pre (mmol/L)	40.71 ± 2.57	40.25 ± 3.09	38.87 ± 1.70	40.04 ± 2.62	0.415
SID post (mmol/L)	41.15 [39.63–43.17]	41.26 [38.80–42.35]	39.37 [36.94–42.53]	40.75 [38.70–41.88]	0.215
SID Δ (mmol/L)	0.78 [−0.33–2.85]	0.80 [0.00–1.75]	1.50 [−1.63–3.01]	−0.32 [−1.03–0.96]	0.613

Normally distributed data are expressed as mean ± standard deviation and were compared with a one-way ANOVA; non-normally distributed data are expressed as median [95% bootstrap confidence interval, 5000 resamples] and were compared with the Kruskal–Wallis test. TR = trocha gait, TRG = trocha & gallop gait, TG = trot & gallop gait.

**Table 5:** Post-exercise biochemical parameters stratified by geographic region (n = 76).

Variable	VC	ANT	*p*-Value	Difference (CI 95%)
pH	7.40 ± 0.04 (n = 34)	7.41 ± 0.05 (n = 38)	0.857	−0.002 [−0.021; 0.018]
CO_2_ (mmHg)	39.95 ± 4.97 (n = 34)	38.90 ± 6.22 (n = 38)	0.434	1.053 [−1.581; 3.686]
HCO_3_^−^ (mmol/L)	24.38 ± 3.19 (n = 34)	23.93 ± 3.46 (n = 38)	0.569	0.450 [−1.113; 2.013]
Na^+^ (mmol/L)	138.00 [137.00–139.00] (n = 34)	137.00 [137.00–138.00] (n = 38)	0.659	1.000 [−0.500; 2.000]
K^+^ (mmol/L)	3.78 ± 0.37 (n = 34)	3.91 ± 0.45 (n = 38)	0.169	−0.137 [−0.331; 0.057]
Ca^++^ (mmol/L)	1.48 [1.41–1.55] (n = 34)	1.50 [1.40–1.55] (n = 37)	0.904	−0.020 [−0.105; 0.110]
Cl^−^ (mmol/L)	101.62 ± 2.53 (n = 34)	102.66 ± 2.50 (n = 38)	0.084	−1.040 [−2.225; 0.145]
Glucose (mmol/L)	116.00 [107.50–126.00] (n = 34)	119.00 [109.50–126.50] (n = 38)	0.973	−3.000 [−14.500; 9.500]
Lactate (mmol/L)	3.25 [1.96–4.30] (n = 34)	3.69 [2.13–4.61] (n = 38)	0.919	−0.440 [−1.995; 1.690]
Creatinine (mmol/L)	1.34 ± 0.21 (n = 34)	1.33 ± 0.23 (n = 38)	0.857	0.009 [−0.094; 0.112]

Comparisons performed using independent *t*-test or Mann–Whitney U test. VC = Valle del Cauca, ANT = Antioquia.

## Data Availability

The original contributions presented in this study are included in the article/Supplementary Materials. Further inquiries can be directed to the corresponding author(s).

## Supplementary Materials

The following supporting information can be downloaded at: https://www.mdpi.com/article/10.3390/ani16172738/s1, Table S1: Exercise-induced change (Δ = post-exercise − pre-exercise) in metabolic and blood-gas variables according to gait modality; Table S2: Omnibus test results for the exercise-induced change (Δ) in metabolic and blood-gas variables among gait modalities; Table S3: Tukey’s HSD post hoc pairwise comparisons for the exercise-induced change in pCO_2_ among gait modalities; Table S4: Dunn’s post hoc pairwise comparisons (Bonferroni-corrected) for the exercise-induced change in HCO3^−^ among gait modalities.

## Abbreviations

The following abbreviations are used in this manuscript:

CCH	Colombian creole horses
VO_2_	Oxygen consumption
pH	Hydrogen potential
HCO_3_^−^	Bicarbonate
pCO_2_	Carbon dioxide partial pressure
