# Supplementary material for: Exercise-Induced Acid–Base, Electrolyte, and Metabolic Responses in Colombian Creole Horses Across Different Gait Modalities
Source: Animals (Basel). 2026 Sep 2;16(17):2738. doi: 10.3390/ani16172738 (PMC13565396; doi:10.3390/ani16172738)
Supplement: Supplementary file 1 [file animals-16-02738-s001.zip › animals-4471589-supplementary.pdf]

Supplementary Materials

Supplementary Table S1. Exercise-induced change (Δ = post-exercise − pre-exercise) in metabolic and blood-gas variables according to gait modality

Variable	FINO	TR	TRG	TG	Statistics
pH	-0.01 ± 0.04 (n=23)	-0.01 ± 0.04 (n=36)	-0.02 ± 0.04 (n=9)	-0.02 ± 0.03 (n=8)	Mean ± SD
CO_2 (mmHg)	0.25 ± 5.34 (n=23)	**-4.38 ± 5.58** (n=36)	-1.39 ± 7.15 (n=9)	-2.19 ± 6.82 (n=8)	Mean ± SD
HCO_3^- (mmol/L)	-0.70 [-1.40--0.15] (n=23)	**-3.50 [-5.43--1.20]** (n=36)	-3.00 [-3.70--1.60] (n=9)	-1.95 [-3.95-0.02] (n=8)	Median [IQR]
Na^+ (mmol/L)	1.00 [0.00-2.00] (n=23)	1.00 [0.00-1.00] (n=36)	0.00 [-1.00-1.00] (n=9)	0.00 [-0.25-2.00] (n=8)	Median [IQR]
K^+ (mmol/L)	-0.13 ± 0.42 (n=23)	-0.11 ± 0.35 (n=36)	-0.08 ± 0.43 (n=9)	-0.05 ± 0.33 (n=8)	Mean ± SD
Ca^{++} (mmol/L)	-0.07 [-0.17-0.02] (n=23)	-0.12 [-0.20--0.07] (n=36)	-0.16 [-0.23--0.10] (n=9)	-0.10 [-0.18--0.03] (n=7)	Median [IQR]
Cl^- (mmol/L)	0.00 [-2.00-1.00] (n=23)	0.00 [-1.00-0.25] (n=36)	-1.00 [-3.00-1.00] (n=9)	0.50 [0.00-1.00] (n=8)	Median [IQR]
Glucose (mmol/L)	2.00 [-5.00-14.00] (n=23)	13.00 [2.75-25.25] (n=36)	-5.00 [-9.00-25.00] (n=9)	14.50 [3.75-30.50] (n=8)	Median [IQR]
Lactate (mmol/L)	1.80 [0.39-3.60] (n=22)	3.64 [0.87-7.33] (n=36)	2.45 [1.35-3.31] (n=8)	2.43 [1.41-4.69] (n=8)	Median [IQR]
Creatinine (mmol/L)	0.14 [0.08-0.23] (n=23)	0.18 [0.04-0.24] (n=36)	0.12 [0.04-0.25] (n=9)	0.20 [0.13-0.30] (n=8)	Median [IQR]

FINO= Paso fino gait; TR= trocha gait; TRG= trocha &gallop gait; TG= trot &gallop gait.

Supplementary Table S2. Omnibus test results for the exercise-induced change (Δ) in metabolic and blood-gas variables among gait modalities

Variable	Applied test	Statistics	p-value	Significative post-hoc comparisons
**pH**	One-way ANOVA	0.13	0.942	—
**CO_2 (mmHg)**	One-way ANOVA	3.05	**0.034 ***	FINO–TR (p=0.021)
**HCO_3^- (mmol/L)**	Kruskal-Wallis	11.93	**0.008 ***	FINO–TR (p=0.003)
**Na^+ (mmol/L)**	Kruskal-Wallis	3.27	0.351	—
**K^+ (mmol/L)**	One-way ANOVA	0.10	0.957	—
**Ca^{++} (mmol/L)**	Kruskal-Wallis	5.52	0.138	—
**Cl^- (mmol/L)**	Kruskal-Wallis	1.52	0.678	—
**Glucose (mmol/L)**	Kruskal-Wallis	5.64	0.131	—
**Lactate (mmol/L)**	Kruskal-Wallis	5.75	0.125	—
**Creatinine (mmol/L)**	Kruskal-Wallis	1.34	0.720	—

FINO= Paso fino gait; TR= trocha gait; TRG= trocha &gallop gait; TG= trot &gallop gait. ** $p < 0.05$*

Supplementary Table S3. Tukey's HSD post hoc pairwise comparisons for the exercise-induced change in pCO2 among gait modalities.

Group 1	Group 2	Difference	CI 95%	Adjusted p-value
FINO	TR	4.626	[0.528 ; 8.723]	**0.021 ***
FINO	TRG	1.637	[-4.399 ; 7.672]	0.892
FINO	TG	2.435	[-3.865 ; 8.736]	0.740
TR	TRG	-2.989	[-8.710 ; 2.732]	0.520
TR	TG	-2.190	[-8.190 ; 3.810]	0.772
TRG	TG	0.799	[-6.660 ; 8.257]	0.992

FINO= Paso fino gait; TR= trocha gait; TRG= trocha &gallop gait; TG= trot &gallop gait.

Supplementary Table S4. Dunn's post hoc pairwise comparisons (Bonferroni-corrected) for the exercise-induced change in HCO_3^- among gait modalities.

Group 1	Group 2	Difference	CI 95%	Adjusted p-value
FINO	TR	3.445	<0.001	**0.003 ***
FINO	TRG	1.649	0.099	0.594
FINO	TG	1.403	0.161	0.964
TR	TRG	-0.727	0.467	1.000
TR	TG	-0.879	0.379	1.000
TRG	TG	-0.150	0.881	1.000

FINO= Paso fino gait; TR= trocha gait; TRG= trocha &gallop gait; TG= trot &gallop gait.
